# High Molecular Weight Hyaluronan Promotes Corneal Nerve Growth in Severe Dry Eyes

**DOI:** 10.3390/jcm9123799

**Published:** 2020-11-24

**Authors:** Gysbert-Botho van Setten, Oliver Stachs, Bénédicte Dupas, Semra Akkaya Turhan, Berthold Seitz, Herbert Reitsamer, Karsten Winter, Jutta Horwath-Winter, Rudolf F. Guthoff, Wolfgang G. K. Müller-Lierheim

**Affiliations:** 1 Department of Clininical Neuroscience, St. Eriks Eye Hospital, Karolinska Institutet, 11282 Stockholm, Sweden; gysbert.van.setten@ki.se; 2 Department of Ophthalmology, University Medical Center Rostock, 18057 Rostock, Germany; oliver.stachs@uni-rostock.de (O.S.); rudolf.guthoff@med.uni-rostock.de (R.F.G.); 3 Quinze-Vingts National Eye Hospital & Vision Institute, 75571 Paris, France; bdupas@15-20.fr; 4 Department of Ophthalmology, Marmara University School of Medicine, 34899 Istanbul, Turkey; semraakkaya85@hotmail.com; 5 Department of Ophthalmology, Saarland University Medical Center, 66421 Homburg/Saar, Germany; berthold.seitz@uks.eu; 6 Department of Ophthalmology & Department of Experimental Ophthalmology and Glaucoma Research, University Clinic Salzburg, Paracelsus Medical University, 5020 Salzburg, Austria; h.reitsamer@salk.at; 7 Institute of Anatomy, Medical Faculty, University of Leipzig, 04103 Leipzig, Germany; kwinter@rz.uni-leipzig.de; 8 Department of Ophthalmology, Medical University Graz, 8036 Graz, Austria; jutta.horwath@medunigraz.at; 9 CORONIS GmbH, 81241 Munich, Germany

**Keywords:** dry eye disease, severe keratitis, diabetes, neuropathic keratopathy, neuropathy, nerve growth, neurotrophic

## Abstract

The purpose of this study was to investigate the effect of high molecular weight hyaluronan (HMWHA) eye drops on subbasal corneal nerves in patients suffering from severe dry eye disease (DED) and to evaluate the damage of subbasal corneal nerves associated with severe DED. Designed as an international, multicenter study, 16 patients with symptoms of at least an Ocular Surface Disease Index (OSDI) score of 33, and corneal fluorescein staining (CFS) of at least Oxford grade 3, were included and randomized into two study arms. The control group continued to use their individual optimum artificial tears over the study period of eight weeks; in the verum group, the artificial tears were substituted by eye drops containing 0.15% HMWHA. At the baseline visit, and after eight weeks, the subbasal nerve plexus of 16 patients were assessed by confocal laser scanning microscopy (CSLM). The images were submitted to a masked reading center for evaluation. Results showed a significant increase of total nerve fiber lengths (CNFL) in the HMWHA group (*p* = 0.030) when compared to the control group, where the total subbasal CNFL did not significantly change from baseline to week 8. We concluded that in severe DED patients, HMWHA from topically applied eye drops could cross the epithelial barrier and reach the subbasal nerve plexus, where it exercised a trophic effect.

## 1. Introduction

Millions of people worldwide are affected by dry eye disease (DED), a heterogeneous, complex disorder of the ocular surface [1]. Within the current concept of a staged treatment, lubricating, hydrating teardrops are the standard long-term therapy for DED [2]. Hyaluronan (HA) eye drops, aiming to increase tear viscosity and enhancing lubrication, are one of the options favored, particularly in Europe and Asia [2]. The combination of concentration and chain length of the HA molecules contained in these eye drops determines their viscoelastic and mucoadhesive properties, resulting in more or less entanglement and rheological synergism with the mucins dissolved in the muco-aqueous layer of the tear film. These physical properties of HA eye drops contribute to minimizing the friction between the moving eyelid and the surface of the eyeball during blinking, thus reducing known stimuli of ocular surface inflammation [3]. High molecular weight hyaluronan (HMWHA) has an anti-inflammatory effect, whereas low molecular weight hyaluronan (LMWHA) promotes inflammation [4,5]. A recent study confirmed in an environmental dry eye stress model in mice that HMWHA eye drops protect the ocular surface from mechanical damage and inflammation better than LMWHA [6]. Future clinical investigations of HMWHA eye drops in humans suffering from chronic ocular surface inflammation should, therefore, include inflammation markers.

The current treatment for severe DED is mainly based on the model of the self-maintaining circle of chronic inflammation [7,8,9,10]. The underlying pathomechanism of severe dry eye disease focuses on inflammation in various situations such as in autoimmune diseases, as well as damage of corneal nerves, for example, in diabetes mellitus or aging [11]. The cornea is by far the most densely innervated tissue of the human body [12]. Nerves provide important trophic support to the corneal epithelium and contribute to ocular surface homeostasis [12,13,14,15,16]. Activated corneal nerves release neuropeptides that contribute to neurogenic inflammation [17,18,19]. Denervation eliminates the neurotrophic support causing neuroparalytic keratitis and breakdown of the corneal epithelium [17,20]. On the other hand, trophic interactions are essential for neuronal survival [21,22,23]. Moreover, there is cross-talk between glia, the extracellular matrix, and neurons [24]. Attrition within the ocular epithelia has been recognized as a lubrication deficit induced factor, enhancing inflammation [25]. Due to these complex interactions, severe DED is regularly associated with compromised corneal nerves [26,27,28,29,30]. This, in turn, results in dysregulation of tear production and blink reflex [31]. Corneal innervation disorders as a primary pathogenic mechanism are due to the absence of ocular pain only diagnosed in a late-stage, although they are often accompanied by keratopathy and delayed epithelial wound healing, sometimes leading to corneal ulcerations and vision loss [32,33,34]. There is a lack of treatments targeting nerve regeneration [34,35].

Patients suffering from neuropathic ocular pain tend to respond poorly to the treatment with lubricant eye drops [36,37]. Experimental evidence suggests that HMWHA, but not LMWHA, can suppress pain in nociceptive afferent nerves [38,39,40], but it is not yet proven whether or not topically applied HMWHA can reduce ocular pain. Moreover, the possible role of hyaluronan in the proliferation of nerve cells has raised attention [24,41,42]. Therefore, we decided to study the potential influence of HMWHA on the corneal nerves within the HYLAN M study. The main intention of the HYLAN M study was to investigate if symptoms and/or signs of patients suffering from severe DED could be improved by substituting the best treatment lubricant eye drops with HMWHA eye drops. In vivo confocal microscopy (IVCM), in particular, when performed as confocal laser scanning microscopy (CSLM), is the gold standard in assessing the subbasal corneal nerve plexus [43,44,45,46]. Within the HYLAN M study, CSLM images were taken at baseline and after eight weeks of treatment and were sent to a masked reading center for evaluation. 

## 2. Experimental Section

### 2.1. Study Design

The HYLAN M study, a multicenter prospective randomized, open-label study, was performed in 11 centers in eight countries. Details have been published elsewhere [47]. The study adhered to the Declaration of Helsinki, was approved by ethics committees of all countries involved, and registered on the database of the European Database for Medical Devices (EUDAMED) under the registration number CIV-16-06-015964.

Patients suffering from severe DED were randomized into two parallel arms. The control group continued with their currently-used therapy by the time of inclusion. In the verum group (Comfort Shield group), the individual lubricant eye drops used by each patient by the time of inclusion were replaced by eye drops containing 0.15% HMWHA (Comfort Shield^®^ eye drops, i.com medical GmbH, Munich, Germany). Concomitant treatment for dry eye, like cyclosporine eye drops, remained unchanged in both arms.

Demographic data and medical history were recorded during the baseline visits. Symptoms and signs associated with DED were assessed at the baseline visit, at week 4, and week 8 follow-up visits, respectively (see Table 1).

The study centers were suggested to optionally take CSLM images at the baseline and week eight visits and provide them to a masked reading center for assessment. Four out of 11 study centers participated in this optional test. These four study centers provided CSLM images of all their per-protocol patients; thus, the electronic randomization used throughout the HYLAN M study also applied to the optional confocal microscopy study. The results of the assessment of the CSLM images of these four study centers are the subject of this report. The results of the other diagnostic tests performed, such as the Ocular Surface Disease Index (OSDI), dropping frequency, best corrected visual acuity (BCVA), corneal fluorescein staining (CFS), tear film break-up time (TBUT), Schirmer 1, tear osmolarity, intraocular pressure (IOP), lid wiper epitheliopathy (LWE), and Yamaguchi score of all 84 per-protocol patients included in the HYLAN M study have been previously reported [47].

### 2.2. Participants

Patients over 18 years suffering from DED of any underlying etiology were eligible for inclusion. The patients had to be under stable, unchanged, dry eye therapy for at least two months (in case of concomitant cyclosporine therapy, three months) by the time of inclusion. Patients were excluded if they participated in any other clinical trial, suffered from eye diseases other than dry eyes, had ocular surgery less than three months prior to study inclusion, were using punctual plugs, or had masquerading conditions as identified by Karpecki [50]. Masquerading conditions are conjunctivochalasis, recurrent corneal erosions, epithelial basement membrane dystrophy, mucus fishing syndrome, floppy eyelid syndrome, giant papillary conjunctivitis, Salzmann’s nodular degeneration, and ocular rosacea.

As inclusion criteria for severe dry eye, the primary criteria, according to Baudouin et al., were chosen [51]. The dry eye symptoms were assessed using the Ocular Surface Disease Index (OSDI) questionnaire, with an OSDI score of 33 or more being required for inclusion [52]. Corneal fluorescein staining (CFS) was selected as a dry eye sign [53]. For inclusion, patients had to have at least one eye with CFS Oxford grade 3 or more, but no confluent CFS. The eyes with the higher staining score were defined as study eyes.

### 2.3. Confocal Scanning Laser Microscopy

The Heidelberg Retina Tomograph (HRT 3), in combination with the Rostock Cornea Module (Heidelberg Engineering GmbH, Heidelberg, Germany), was used for the in vivo assessment of the corneal subbasal nerve plexus (SNP), as described previously [54,55]. Both eyes were anesthetized with topical anesthetic and covered with artificial tears. To prevent eye movements, the patients were asked to fixate on a spotlight with the unexamined eye.

**Figure 1:**
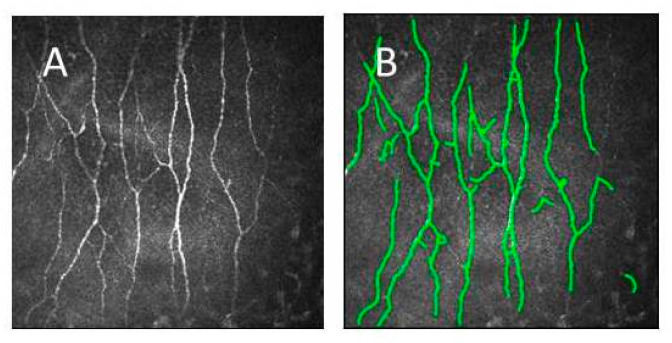
Single image from the subbasal nerve plexus (SNP) in an individual (**A**) and automatically detected nerve fibers used for quantification (**B**).

Five non-overlapping images were taken in the central region of the cornea, close to the apex and more than 0.5 mm apart from the inferior whorl (see Figure 1A for an example of an image and Figure 1B after image processing by the reading center).

Image processing and quantitative image analysis were performed by the reading center using Mathematica (Version 11.3, Wolfram Research Inc., Champaign, IL, USA), as previously described [56]. The following SNP parameters were calculated: corneal nerve fiber length (CNFL), defined as the total length of all nerve fibers per unit area (mm/mm^2^); corneal nerve fiber density (CNFD), defined as the number of nerve fibers per unit area (n/mm^2^); corneal nerve branch density (CNBD), defined as the number of branching points per unit area (n/mm^2^); average weighted corneal nerve fiber tortuosity (CNFTo), reflected variability of nerve fiber directions and defined as absolute nerve fiber curvature/nerve fiber length (μm^−1^); corneal nerve connection points (CNCP), defined as the number of nerve fibers crossing the area boundary (connections/mm^2^); average corneal nerve single-fiber length (CNSFL), defined as the average length of nerve fibers (μm); and average weighted corneal nerve fiber thickness (CNFTh), measured as mean thickness perpendicular to the nerve fiber course (μm).

### 2.4. Statistical Analysis

Statistical analysis was performed using IBM SPSS Statistics (Version 22, IBM Corp., Armonk, New York, NY, USA). Descriptive statistics were calculated, and box plots were generated. Data were examined for normal distribution using the Shapiro–Wilk test. Group comparisons were performed using the Wilcoxon Signed Rank Test and the Mann–Whitney U test, respectively. The significance level was determined to be *p* < 0.05.

## 3. Results

### 3.1. Participant Demography

Table 2 contains the socio-demographic characteristics of the patients with the CSLM assessment of the SNP.

### 3.2. Confocal Microscopy Results

**Figure 2:**
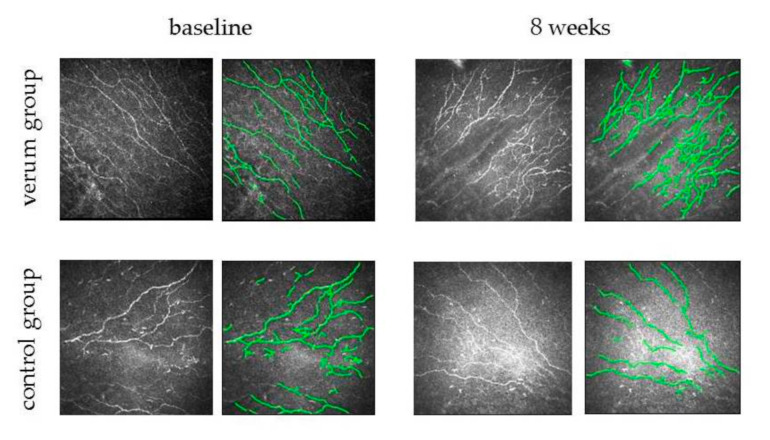
Typical SNP images of subjects from the control and study group, as well as a schematic representation of detected nerve fibers used for characterization of the SNP at baseline and after 8 weeks of treatment.

Five CSLM images of eight patients of the control group and eight patients of the Comfort Shield group taken at the end of the baseline visit and at the end of the week 8 visit were analyzed (see examples in Figure 2).

**Figure 3:**
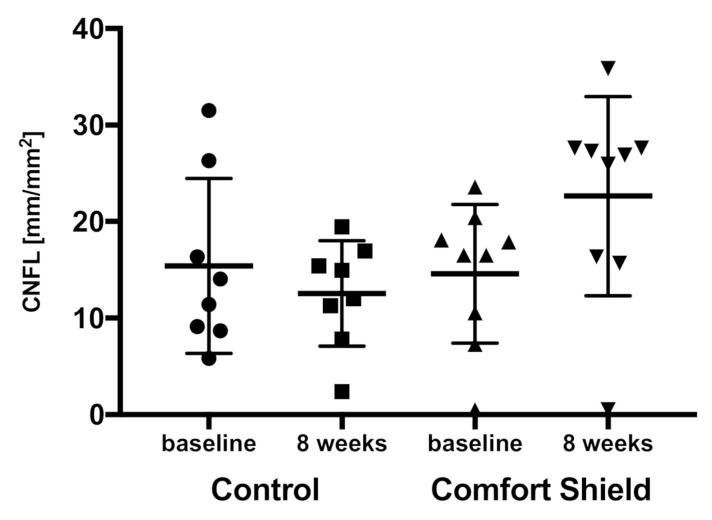
CNFL of the Comfort Shield group and the control group at baseline and eight weeks.

We found a statistically significant difference in CNFL between baseline and the eight weeks follow-up visit; the Comfort Shield group showed a significant difference in CNFL (*p* = 0.030) contrary to the control group (*p* = 0.294). CNFL was comparable for Comfort Shield and control at baseline (*p* = 0.793) and showed a significant difference after eight weeks (*p* = 0.031). Possibly due to the small number of patients, we did not find significant differences for the other SNP parameters (CNFD, CNBD, CNFTo, CNCP, CNSFL, CNFTh). Moreover, patients suffering from severe dry eye generally do not have a well-developed SNP, and there was a lot of foreign tissue in the vicinity of the SNP that complicated the image analysis. Figure 3 summarized the CNFL findings of the Comfort Shield group and the control group at baseline and eight weeks visit.

## 4. Discussion

Due to the heterogeneous, multicausal nature of DED, particularly in patients suffering from severe, chronic DED, a personalized clinical management resulting in an individualized optimum therapy is required [57]. Only patients under stable therapy had been included in the HYLAN M study, and their optimum individual therapy served as a control in comparison to patients in which 0.15% HMWHA eye drops were tested. The assessment of the subbasal corneal nerve plexus was an optional test in addition to the standard diagnostic test battery of the study. Four out of 11 study centers provided CSLM images from 16 per-protocol patients, eight each in the Comfort Shield group and in the control group. The SNP is usually not well structured in severe dry eye disease [58]. Due to the small number of patients, only the results of CNFL showed a significant difference between the two study arms. This is in accordance with other studies reporting that CNFL is the most reproducible parameter in the evaluation of IVCM images of the subbasal nerve plexus [28,59,60,61,62,63,64,65]. 

Until recently, HA eye drops had been applied as a lubricating, hydrating, and mechanically buffering tear substitute [66]. It was known that the apical surface of the superficial epithelial cells of the cornea and conjunctiva have HA receptors (CD44 and HARE), which can bind HA and thus support the antiadhesive properties of the glycocalyx [67,68,69,70,71]. HMWHA, but not LMWHA, can also adhere to the membrane-bound mucins of the glycocalyx, thus strengthening the cellular barrier of the ocular surface [72]. HA is an essential part of the extracellular matrix (ECM) and plays an important molecular weight dependent role in wound healing and immunoregulation [4,73,74,75,76,77,78]. Disturbed immunoregulation involving chronic inflammation, which triggers a vicious circle, is currently considered the characteristics of severe DED [10]. HMWHA enables cross-bridging between the HA receptors of adjacent cells and can thus contribute to the mechanical stabilization of the wing cell layers of the corneal epithelium [79,80,81]. Reactive oxygen species (ROS) formed during inflammatory processes effectively cleave HMWHA, which in turn enhances the inflammatory process and weakens the cross-bridging function of HA between epithelial cells [82]. So far, it had been unknown whether HMWHA from topically applied eye drops can, in a situation of chronic ocular inflammation, pass the ocular surface barrier to recover the homeostatic HA weight distribution in the extracellular matrix. The first evidence came from an animal experiment where 0.15% HMWHA eye drops were compared with 0.1% and 0.3% LMWHA eye drops with respect to their ability to prevent and treat DED caused by environmental stress [6]. The 0.15% HMWHA eye drops proved superiority with respect to the prevention and treatment of inflammation and stabilization of aqueous tear secretion and mucin production [6].

The HYLAN M study indicated that topically applied HMWHA could pass the intercellular barrier of the corneal epithelium. By changing the extracellular matrix in the proximity of the subbasal nerve plexus, this could result in trophic effects reflected in the significant regeneration of compromised nerves. This also provided evidence that HMWHA becomes available in the ECM in all cell layers of the corneal epithelium and thus can contribute to regaining ocular surface homeostasis in eyes with chronic inflammation. Further methods detecting specific anti-inflammatory and neurotrophic factors such as nerve growth factor (NGF) in the tear film or in the ocular surface will provide valuable additional information in future clinical studies. On the other hand, the study showed that within eight weeks of treatment, simultaneously with nerve growth, the symptoms of patients with severe DED improved significantly. According to the results of the HYLAN M study, in combination with the animal study [6], we may conclude that 0.15% HMWHA eye drops grant a holistic approach in the treatment of DED, simultaneously addressing the various and complex interacting pathomechanisms of the disease: lubrication, hydration, stabilization of glycocalyx and barrier function, downregulation of inflammation, trophic support to corneal nerves, increasing goblet cell counts and expression of MUC5AC [83], support of aqueous tear production, and reduction of pain.

It needs to be emphasized that the effect of HMWHA on nerve regeneration has only been investigated in a very small number of eyes. Hence, conclusions on significance in numbers cannot be given. Nevertheless, the high incidence of nerve regeneration during treatment with HMWHA was clearly different from the unchanged situation in the control group. Future studies with a higher number of eyes and a primary focus on nerve regeneration will provide further details.

As the HYLAN M study included dry eye patients with any disease etiology, it seems likely that patients with corneal nerve injury or degeneration as an underlying cause for ocular surface disease or neurotrophic keratopathy would benefit from treatment with HMWHA eye drops [11,84]. The causes may include acute nerve injury like in ocular surgery, refractive surgery, corneal cross-linking, chemical burns, or ocular trauma [85,86]. Similarly, HMWHA eye drops may also be effective in promoting neuroregeneration in progressing peripheral neuropathies associated with ocular infections, keratoconus, small-fiber neuropathy, diabetes mellitus, or simply aging [87,88,89,90]. 

The progressive loss of corneal sensory innervation of any etiology may result in neurotrophic keratopathy (NK) [15,16,35,91,92,93,94]. NK is characterized by corneal anesthesia and is a condition that is very difficult to treat, especially as for the required regeneration of trigeminal terminal nerve fibers, no such treatment is currently available [35,93,95]. Medical management with lubricating eye drops, anti-inflammatory agents, and anti-proteases provide unspecific temporary relief in NK but do not prevent disease progression [93,96]. Whereas some degree of inflammation promotes nerve regeneration, excessive inflammation may lead to a loss of corneal innervation and subsequent development of NK [15]. As corneal nerve regeneration and inflammation are intertwined, the therapeutic strategy must consider the interaction of both pathways [15]. HMWHA eye drops seem to offer a promising treatment option in this situation.

According to the International Diabetes Federation (IDF), more than 400 million people worldwide suffer from diabetes mellitus (DM). DM is associated with a progressive loss of peripheral nerves. Corneal nerve damage may serve as an early indicator in DM [63,97,98,99]. The prevalence of corneal neuropathy in diabetic patients is approximately 50% [32,100,101,102,103]. However, corneal neuropathy, as a manifestation of DM, is underrated due to the absence of ocular discomfort and pain [33,98,103,104]. Persistent corneal epithelial erosions, superficial punctate keratopathy, delayed epithelial regeneration, and decreased sensitivity are associated with diabetic keratopathy [32,34,105,106]. Diabetic keratopathy is a significant clinical problem and a progressing disease, and currently, no effective treatment is available [34]. IVCM has proven to be a valuable and reliable diagnostic tool to assess nerve fiber damage and assess improvement of risk factors for diabetic neuropathy, thus allowing visualizing treatment success [59,107,108]. Having shown that 0.15% HMWHA eye drops support corneal nerve regeneration allows the assumption that these eye drops will also prove as an effective preventive therapy against the progression of diabetic keratopathy. This may contribute to lower the enormous global economic burden of DM [109]. The results reported here were obtained from a small number of patients. This report is, therefore, intended to encourage further clinical research rather than to provide comprehensive answers or interpretation.

## 5. Conclusions

This is the first evidence that topically applied HMWHA eye drops induced a significant neurotrophic effect on the subbasal nerve plexus in humans. When applied after any kind of ocular surgery, HMWHA eye drops may serve to support the recovery of damaged nerves. Moreover, HMWHA eye drops offered a new therapeutic option in preventing and treating ocular surface disease, in particular diseases associated with nerve damage like diabetic keratopathy and all forms of neuropathic keratopathy. Future research will focus on the question if patients with diabetic keratopathy and other forms of neuropathic keratopathy could benefit from HMWHA eye drops. HMWHA eye drops provide a holistic approach while simultaneously addressing different interacting pathomechanisms of DED.

## Figures and Tables

**Table 1:** Diagnostic testing schedule with optional tests in round brackets.

Test	Baseline	Week 4	Week 8
OSDI	X	X	X
Dropping frequency	X	X	X
BCVA	X	X	X
CFS	X	X	X
TBUT	X	X	X
Schirmer 1	X		X
Tear osmolarity	X		X
IOP	X		X
LWE, Korb score [48]	(X)		(X)
Yamaguchi score [49]	(X)		(X)
Confocal microscopy	(X)		(X)

Abbreviations: Ocular Surface Disease Index (OSDI), best corrected visual acuity (BCVA), corneal fluorescein staining (CFS), tear film break-up time (TBUT), intraocular pressure (IOP), and lid wiper epitheliopathy (LWE).

**Table 2:** Socio-demographic characteristics according to the treatment arm (*n* = 16).

		Comfort Shield *n* = 8	Control *n* = 8
Age (years)	n	8	8
	mean (SD)	59.5 (9.2)	61.6 (18.4)
	min, max	36, 77	47, 73
Sex n (%)	n	8	8
	female	6 (75)	6 (75)
	male	2 (25)	2 (25)
Medical History	n	8	8
	Sjögren syndrome	2	3
	rheumatoid disease	3	2
	rheumatoid + thyroid disease	1	
	thyroid disease		1
	Graves disease + betablocker	1	
	diabetes mellitus + betablocker		1
	no dry eye related disease	1	1
